# AIM2 and Psoriasis

**DOI:** 10.3389/fimmu.2023.1085448

**Published:** 2023-01-18

**Authors:** Yuxi Zhang, Xiaoqing Xu, Hui Cheng, Fusheng Zhou

**Affiliations:** ^1^ Department of Dermatology, The First Affiliated Hospital, Anhui Medical University, Hefei, Anhui, China; ^2^ Institute of Dermatology, Anhui Medical University, Hefei, Anhui, China; ^3^ Key Laboratory of Dermatology (Anhui Medical University), Ministry of Education, Hefei, Anhui, China; ^4^ Inflammation and Immune Mediated Diseases Laboratory of Anhui Province, Hefei, Anhui, China

**Keywords:** psoriasis, AIM2, inflammasome, keratinocytes, epigenetic, trained immunity

## Abstract

Psoriasis is a chronic inflammatory skin disease occurring worldwide, with multiple systemic complications, which seriously affect the quality of life and physical and mental health of patients. The pathogenesis of psoriasis is related to the environment, genetics, epigenetics, and dysregulation of immune cells such as T cells, dendritic cells (DCs), and nonimmune cells such as keratinocytes. Absent in melanoma 2 (*AIM2*), a susceptibility gene locus for psoriasis, has been strongly linked to the genetic and epigenetic aspects of psoriasis and increased in expression in psoriatic keratinocytes. AIM2 was found to be activated in an inflammasome-dependent way to release IL-1β and IL-18 to mediate inflammation, and to participate in immune regulation in psoriasis, or in an inflammasome-independent way by regulating the function of regulatory T(Treg) cells or programming cell death in keratinocytes as well as controlling the proliferative state of different cells. AIM2 may also play a role in the recurrence of psoriasis by trained immunity. In this review, we will elaborate on the characteristics of AIM2 and how AIM2 mediates the development of psoriasis.

## Introduction

Psoriasis is a chronic systemic inflammatory skin disease, as well as a polygenic genetic disorder, the etiology of which is not completely clear (1, 2). Psoriasis invades most regions and races worldwide, affecting up to 125 million individuals (3). People with psoriasis are more likely to suffer from Crohn’s disease (4), cardiovascular disorders (5), psoriatic arthritis (6), depression (7), cancer (8) and obesity (9). The most common type of psoriasis is psoriasis vulgaris, which clinically features pink or red plaques distinct from normal skin and covered with white scales, often symmetrically distributed on the elbows, calves, scalp, abdomen, and back (10). Psoriasis is susceptible to recurrence and requires long-term treatment; many patients suffer from a huge physical and mental burden, as well as an enormous financial burden.

Psoriasis is considered to be associated with environmental factors, genetic factors, and disturbances between immune and nonimmune cells, and in recent years, epigenetic regulatory mechanisms have also been found to play a significant role in development of psoriasis (11, 12). Psoriasis is often easily irritated by factors such as injury, drugs, infections, stress, or other stimuli (13). Unlike Mendelian diseases, psoriasis is a complex polygenic genetic disease that often presents the characteristics of family aggregation. Through several studies researchers identified the importance of the psoriasis susceptibility gene 1 (PSORS1), which was the main susceptibility locus of psoriasis, accounts for 35-50% of disease heritability (14, 15). More than 40 susceptibility loci for psoriasis have been identified *via* genome-wide association studies (GWASs) and immunochip studies (16). The IL-23/IL-17 axis is the most important part of the immunomodulatory mechanism in psoriasis, especially IL-17, which plays a central role in disease progression (17). Besides, other cytokines are also actively involved in the immune regulation of psoriasis. Among them, IL-18 expression is significantly correlated with the severity of psoriasis (18). IL-23 binding to IL-1β or IL-18 promotes IL-17 production (19, 20). IL-1β and IL-23 can also activate γδ T cells and amplify T helper cell 17 (Th17) responses, while IL-18 reacts synergistically with IL-23 to similarly activate γδ T cells and induce Th1 immune responses (20, 21).

In addition to the above influencing factors, the inflammasome also participates in the pathogenesis of psoriasis (22). AIM2 polymerizes with other proteins to form the AIM2 inflammasome, which is one of the inflammasomes closely associated with psoriasis (22). The AIM2 inflammasome was found to be expressed increasingly in psoriatic skin, especially in keratinocytes (23). In addition to the role of the AIM2 inflammasome, AIM2 inflammasome-independent way may also play a significant role in the pathogenesis of psoriasis. For example, a recent study showed that AIM2 expression in Treg cells contributes to restraining the autoimmunity response (24).

In this review, we focus on the characterization of AIM2 and its relationship with psoriasis, how AIM2 plays an inflammasome-dependent and possibly inflammasome-independent role in the development of psoriasis, and how AIM2 may potentially modulate psoriasis relapse by training immunity.

## AIM2 inflammasome

Since Martinon et al. (25) came up with the concept of the inflammasome in 2002, it has been described in many immune-related diseases, acting as an important part of innate immunity. Inflammasomes are intracellular multiprotein complexes (26). When encountering dangers such as microbes or self-DNA, the inflammasome assembles under the guidance of its specific pattern-recognition receptors (PRRs) to respond to pathogen-associated molecular patterns (PAMPs) (27). PAMPs include cell wall components of microorganisms, microorganisms, or nucleic acids. PRRs consist of PRRs localized on the plasma membrane and PRRs localized in the cytoplasm, while absent in melanoma 2-like receptors (ALRs) are intracytoplasmic PRRs that specifically recognize PAMPs in the cytoplasm (28).

**Figure 1:**
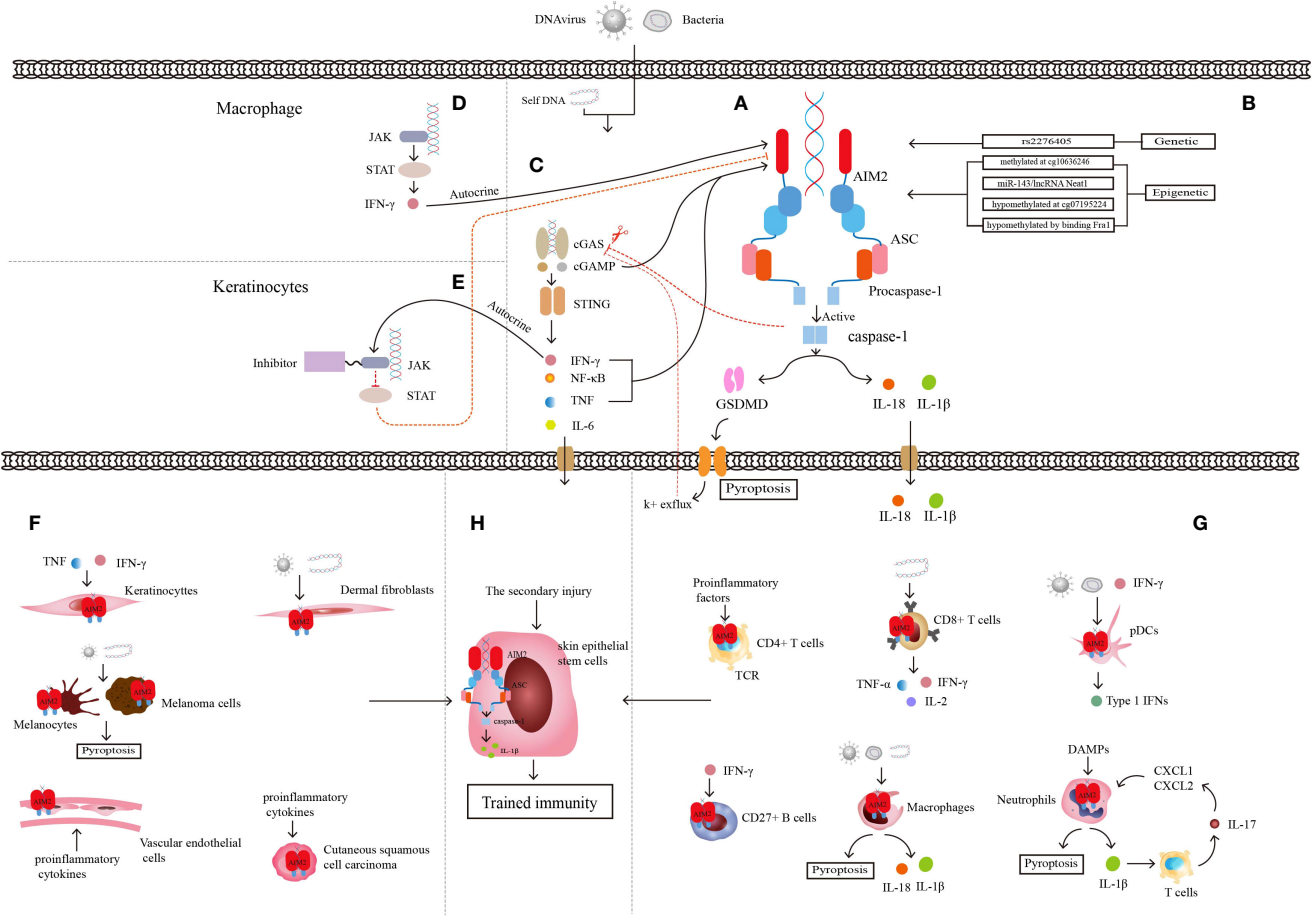
Characteristics of AIM2 inflammasome. **(A)** Structure of AIM2 inflammasome. **(B)** Genetic regulation and epigenetic regulation of AIM2. **(C)** Relationship between cGAS-STING pathway and AIM2 inflammasome. **(D)** Relationship between JAK-STAT pathway and AIM2 inflammasome in macrophages linked by autocrine secretion of IFN-γ. **(E)** Relationship between JAK-STAT pathway and AIM2 inflammasome in keratinocytes after JAK inhibitor application. JAK-STAT and cGAS-STING pathway are linked through the autocrine of IFN-γ. **(F)** Some non-immune cells mediating inflammation through AIM2 inflammasome. **(G)** Some immune cells mediating inflammation through AIM2 inflammasome. **(H)** Skin epithelial stem cells are involved in trained immunity through AIM2 inflammasome and the downstream inflammatory cytokine IL-1β. AIM2 integrates the trained immune memory properties of immune cells with non-immune cells.

AIM2 is a cytoplasmic dsDNA sensor, and an initial member of the ALRs family for inflammasome sensors (29, 30). AIM2 belongs to the PYHIN family and has one or two hematopoietic, interferon-inducible and nuclear (HIN) domains at the C-terminus that recognize and bind to cytoplasmic DNA, and a Pyrin domain (PYD) at the N-terminus that interacts with the PYD domain of the adapter protein, which called apoptosis-associated speck-like protein containing a CARD(ASC) (31, 32). ASC binds to procaspase-1 through CARD-CARD homologous protein interaction and completes the assembly of the AIM2 inflammasome complex (31). The caspase-1 generated by cleavage after procaspase-1 activation cleaves gasdermin-D (GSDMD) and inserts its N-terminal into the cell membrane to form a hole that cytokines can be released through, and the hole made of GSDMD insertion could result in a special cell death named pyroptosis and further exacerbate inflammation (33). Activated caspase-1 cleaves proIL-1 and proIL-18, and leading to the activation of downstream IL‐1β and IL‐18 and an inflammatory response (**Figure 1A**) (34–36). IL-18 is a member of the IL-1 family, takes part in immune cells such as Th1, Th2, natural killer (NK) cells, IL‐17‐producing γδ T cells, and macrophage activation, and acts as an IFN γ‐inducing factor to activate NF-κB to trigger inflammation (37). In human skin, the accumulated DNA in the cytoplasm of keratinocytes activates the AIM2 inflammasome and leads to the release of IL-1β, and keratinocytes are recognized as the main source of IL-1β (38). IL-1β is increased in keratinocytes of psoriatic skin and promotes the development of T-cell-dependent skin inflammation (39).

AIM2 can only be bound by dsDNA of a suitable length. If the length of dsDNA is less than 80bp, AIM2 cannot effectively oligomerize around it, so the inflammasome cannot be activated by the short dsDNA, and AIM2 may only play a regulatory role under this circumstance (40, 41). The activated AIM2 inflammasome plays an important role in the occurrence and development of tumors, the innate immune response, proliferation and apoptosis of cells, and neurodegenerative disorders (30, 42).

## Genetic and epigenetic control of the Aim2 inflammasome

Mutation of the *AIM2* gene often drives the development of different diseases. The stop-gain variant in the *AIM2* gene, whose definite site is rs2276405 (**Figure 1B**), is significantly enriched in psoriasis (43). Methylation of the *AIM2* gene at the site cg10636246 drives the relationship between trauma exposure, posttraumatic stress disorder (PTSD), the level of C-reactive protein (CRP) and other inflammatory mediators, such as IL-6, IL-10, and TNF-α, in the body (44, 45). MicroRNA (miRNA), a non-coding RNA (ncRNA) of approximately 22 nucleotides in length, binds to messenger RNA (mRNA) targets of inflammasome genes for post-transcriptional regulation of inflammasome expression (46). Researchers transfected microRNA-143 (miR-143) (**Figure 1B**), which has been shown to target several inflammation-related genes, into the Jurkat cell line and found significant increases in mRNA expression of both AIM2 and ASC (47). Another type of ncRNA, long noncoding RNA (lncRNA), which is defined as untranslated RNA more than 200 nucleotides in length, can regulate several specific cis- or trans-acting elements (48). Researchers have identified nuclear enriched abundant transcript 1 (Neat1), a kind of lncRNA that can be transcribed from multiple endocrine tumor loci. The lncRNA Neat1 (**Figure 1B**) can reduce the inflammatory response by promoting the assembly of AIM2, activating the oligomerization of caspase-1, and producing cytokines (49).

In addition to AIM2, researchers have explored a 600-bp-long CpG island exists in the transcription initiation site of the ASC gene named *PYCARD/TMSI*, the methylation state of this CpG island is associated with the expression level of ASC. The unmethylated CpG site signifies the active transcription of ASC, the hypermethylated site indicates the inactive state, while the aberrant methylation of that is associated with gene silence (50, 51). Some experiments also proved that ASC methylation affects inflammasome activation (52).

## Aim2 inflammasome-related signals

AIM2 can also interact with multiple signaling pathways. The Janus kinase/signal transduction and activator of transcription (JAK/STAT) signaling pathway is strongly linked to immune-mediated diseases, and the mutation and polymorphisms of JAK and STAT genes result in several inflammatory and autoimmune diseases, include psoriasis (53). The mouse experiments with the atherosclerotic cardiovascular disease model show that JAK/STAT mutation led to a series of changes within cells, such as metabolic disorder, abnormal proliferation, DNA oxidative damage, and replication error. The final result is activation of the AIM2 inflammasome and the production of mature IL-1β and IL-18 (**Figure 1D**) (54). JAK-STAT signaling expression is upregulated in lupus erythematosus skin lesions, and the application of JAK1 inhibitors significantly reduces the expression of proinflammatory mediators such as AIM2 and improves skin lesions in lupus mouse models (55). Quercetin was found to reduce caspase-1 expression and aggregation of the AIM2 inflammasome and the production of IL-18 and IL-1β by inhibiting IFN-induced JAK-STAT expression in human keratinocytes, thus reducing skin inflammation (**Figure 1E**) (56).

## Activation of the AIM2 inflammasome in nonimmune cells

Keratinocytes are among the components that form the physical barrier of the skin and regulate the immune response of the skin microenvironment through the secretion of proinflammatory cytokines, chemokines, antimicrobial peptides (AMPs) and growth factors (57). Keratinocytes directly or indirectly crosstalk with immune cells through these proinflammatory cytokines and chemokines and can also express related receptors to recruit immune cells to secrete more proinflammatory ingredients, leading to immune disease (**Figure 1F**) (58). Keratinocytes detect a wide range of PAMPs by expressing abundant PRRs (23, 59), including AIM2 (60). Although researchers found that AIM2 is expressed only in the Langerhans cells and melanocytes of healthy skin, AIM2 can be upregulated significantly in keratinocytes when skin suffers from harmful conditions such as psoriasis or wounds. AIM2 and its downstream cytokine IL-1β always play an important role in the immune defense response of keratinocytes, and keratinocytes are considered to be the main source of IL-1β in skin cells (59). Although the cytosolic DNA receptor cyclic GMP-AMP synthase and the stimulator of interferon genes (cGAS-STING) is also a DNA recognition receptor, only AIM2 can trigger the secretion of IL-1β in keratinocytes (61). When keratinocytes are infiltrated with proinflammatory cytokines such as IFN-γ and TNF-α, AIM2 inflammasome activation and downstream IL-1β release have been proven to result in psoriasis lesions (62).

Arboviruses induce the expression of the AIM2 inflammasome in human dermal fibroblasts, and the inhibition of caspase-1 restrains the effect of AIM2 but enhances viral replication, showing that the function of dermal fibroblasts is positively associated with early infection through AIM2 (63). Researchers used the EP1 pulse protocol to observe the expression of cytokine mRNA in mouse skin after pDNA electrotransfer for four hours, and as they expected, the expression of AIM2 in both fibroblasts and keratinocytes was upregulated, describing the role of the DNA recognition receptor of AIM2 in fibroblasts (64).

Skin epithelial stem cells, which contribute significantly to skin wound healing, were first discovered to have the function of inflammatory memory as nonimmune cells. This trained immunity is mediated by the AIM2 inflammasome. Researchers believe that recurring immune skin diseases such as psoriasis may be associated with AIM2-mediated inflammatory memory in skin epithelial stem cells (65).

Although scientists have found that AIM2 activation in keratinocytes drives the development of many immune diseases, some other skin cells have the opposite expression; for example, AIM2 is restrictedly expressed in melanocytes in normal skin (23). Wang and his colleagues also proved that the viral dsDNA analog could activate AIM2 and then induce apoptosis of primary cultured normal human melanocytes. Knockdown of AIM2 can partially reduce apoptosis of melanocytes, and the reason may be related to the low expression of AIM2 in melanocytes (66). However, AIM2 plays an immunosuppressive role in the melanoma microenvironment in mouse model based on that AIM2-dependent IL-1β and IL-18 promote tumor growth and that AIM2 acts as a negative regulator of the STING pathway, inhibiting STING-mediated type I IFN secretion and migration of CD8+ T cells to tumors (67).

Cutaneous squamous cell carcinoma originates from skin keratinocytes, and chronic inflammation is one of the important pathogenic risk factors. The protein and mRNA expression of AIM2 is increased in cutaneous squamous cell carcinoma cells, and AIM2 was discovered to possibly be involved in the regulation of the cell cycle as well as cell survival and apoptosis (68).

Vascular endothelial cells also express AIM2 and respond to inflammation by upregulating AIM2 expression. For mice of acute or chronic vascular injury, activation of AIM2 leads to limited endothelial cell regeneration (69). Inhibition of AIM2 also reduced IL-1β and IL-18 expression levels in vascular endothelial cells in atherosclerosis-prone mouse model (70).

## Activation of the AIM2 inflammasome in immune cells

Overactivation or suppression of the innate and adaptive immune systems are considered one of the pathogeneses in immune-mediated diseases, and psoriasis is one of the diseases in which immune cells play an important role. Psoriasis-associated immune cells, including DCs, monocytes, macrophages, neutrophils, mast cells, and T cells, play a central role in the development and progression of the disease (71). The comprehensive analysis of core genes and innate immune cells in psoriasis shows that AIM2 has a positive correlation with activated dendritic cells and M1 macrophages, as well as a negative correlation with resting mast cells (72), suggesting a close association of AIM2 inflammasomes with immune cells (**Figure 1G**) (**Table 1**).

**Table 1:** The activation of AIM2-inflammasome in immune cells.

Cell types	Role of AIM2 inflammasome in immune cells	Reference
CD4+T cells	AIM2 is expressed in both human and mouse CD4+ T cells and is regulated by TCR activation, but has no specific effect on Th-cell differentiation and more negatively regulates the differentiation and stabilization of FOXP3+ Treg cells in a inflammasome-independent manner	(73, 74)



	Inflammatory microenvironment in patients with abdominal aortic aneurysm leads to upregulation of AIM2 expression in CD4+ T cells	

CD8+T cells	AIM2 can be used as an adjuvant for DNA vaccines in viral infections or tumors, then recognizes DAMPs, produces inflammatory cytokines, and enhances the proliferation and function of CD8+ T cells	(75–77)


B cells	AIM2 can be activated by IFN-γ but not dsDNA in adult mature CD28+ B cells	(78)

	AIM2 can be stimulated by synthetic dsDNA leading to IL-1β secretion by primary B cells cultured *in vitro*, but caspase-1 remained at low activity levels	


Dendritic cells	AIM2-mediated activation of caspase-1-dependent inflammasome signaling and production of IL-18 and IL-1β affect the host immune response to malaria infection, and the response of AIM2 to inflammation can be combined with the IFN produced by pDCs	(79–82)


	AIM2 is activated by type I IFN, produces IL-10 and IL-1α *via* caspase-1, leading to immunosuppressive properties of pDCs and favoring lung cancer progression	


	Human adenoviruses, *Francisella tularensis*, vaccinia virus, mouse cytomegalovirus, and other pathogens respond to AIM2 within DCs, leading to AIM2 and caspase-1-dependent activation of inflammasome signaling pathways and IL-18 and IL-1β production	



Macrophages	Cytoplasmic DNA from pathogens, organelle responds to AIM2, prompting macrophages to secrete IL-18 and IL-1β, contributing to host defense and inducing Th17 adaptive immune responses	(83, 84)


	dsDNA triggers caspase-1-mediated AIM2-ASC-dependent apoptosis and drives tumor cell apoptosis	

Neutrophils	Neutrophils express high levels of AIM2, ASC, caspase-1 and IL-1β, and AIM2 can induce GSDMD pyroptosis in neutrophils	(85–87)

	Infection cells release IL-1β through AIM2 inflammasome signaling pathway, and upregulates IL-17 secreted by Th17 cells, which drives chemokines and recruits more neutrophils into the inflammatory microenvironment	



AIM2, absent in melanoma 2; TCR, T-cell receptor; Th cells, helper T cells; IL, interleukin; IFN‐γ, interferon γ; FOXP3, transcription factor forkhead Box P3; DCs, dendritic cells; DAMPs, damage-associated molecular patterns; ASC, apoptosis-associated speck-like protein containing a carboxy-terminal CARD.

AIM2 was found to be expressed in CD4+ T cells in both humans and mice and regulated by T-cell receptor (TCR) activation, but there were no significant changes in the levels of differentiated Th1, Th2, and Th17 cells from CD4+ T cells in AIM2-knockout mice, and the expression of specific cytokines secreted by these cells was also not altered significantly. *In vitro* cellular experiments demonstrated that AIM2 is not involved in the differentiation of these T-cell subpopulations. CD4+ T cells can differentiate into Th-cell subpopulations and Treg cells, but since ASC deficiency cannot affect transcription factor forkhead Box P3 (FOXP3) production or differentiation of FOXP3+ Treg cells, AIM2 cannot regulate Treg cells in an inflammasome-dependent way (73). Researchers also found that various proinflammatory factors in patients with abdominal aortic aneurysms cause an imbalance of CD4+ T cells and upregulate the expression of AIM2 in them (74). AIM2 can be added as an adjuvant to DNA vaccines to mediate caspase-1 activation and IL-1β and IL-18 production in mouse models, as well as pyroptosis, to release damage-associated molecular patterns (DAMPs) and transmit inflammatory signals to surrounding cells (75). This kind of vaccine would enhance the induction of multifunctional CD8+ T cells, which express IL-2, TNF-α, and IFN-γ, and the vaccines have been proven to be effective in preventing virus-induced myocarditis in a mouse model (76). Moreover, this special vaccine with AIM2 as the adjuvant can also activate the proliferation of mouse CD8+ T cells and enhance their antitumor activity (77).

Analysis of adult blood and cord blood revealed that AIM2 is preferentially expressed in adult mature CD27+ B cells and that AIM2 activation is dependent on IFN-γ rather than IFN-α and synthetic dsDNA. Human primary B cells cultured *in vitro* can be stimulated by synthetic dsDNA to release IL-1β, but caspase-1 remains at low activity levels. The regulatory expression of AIM2 in human B cells cultured *in vitro*, human *in vivo* B cells, and mouse B cells is still not fully understood, since part of the regulatory effects is the opposite (78).

Monocytes can differentiate into macrophages that remain in tissue and dendritic cells that roam around the tissue as prototypical antigen-presenting cells, and both can discriminate, phagocytose and present microbial and foreign matter (88–90). There are three specific cutaneous DC populations, pDCs, dermal myeloid DCs (mDCs), and epidermal Langerhans cells (LCs), each of which appears to have a significant impact on inflammatory skin disease. These cells can act as antigen-presenting cells or an important cellular source of pathogenic cytokines, but only pDCs can activate AIM2, which mediates disease progression in an inflammasome-dependent manner (91, 92). In malaria, deficiency of AIM2 or caspase-1 significantly reduces IL-1β production in pDCs, conventional DCs (cDCs), and macrophages, and type I IFN regulates inflammasome activation and IL-1β production, as we all know that pDCs are a major source of type I IFN so that the AIM2 inflammasome response to infection can then be cross-linked to type I IFN signaling (79). In lung cancer tissues, pDCs have been found to activate the AIM2 inflammasome through type I IFN signaling, conducive to caspase-1 aggregation and the production of IL-1α and IL-10 but not IL-1β and IL-18 indirectly, leading to the immunosuppressive properties of pDCs and thus benefiting tumor progression (80). Previous studies confirmed that many pathogens could respond to AIM2 activation in DCs and macrophages of human and mouse and induce AIM2 inflammasome cleavage of mature proIL-1β and proIL-18 in succession (81, 82).

Macrophages can form the first line of defense to protect the body from harmful substances *via* antigen presentation, phagocytosis, and inflammasome activation (93, 94). During the inflammatory response, many cytokines, such as IL-6, IL-1β, TNFα (83), and excessive reactive oxygen species (ROS) (95), are released from activated cultured macrophages, which can activate the AIM2 inflammasome (96). In contrast, when the AIM2 inflammasomes from human or mouse immune cells encounter dsDNA from microorganisms, other organelles, and extracellular DNA, macrophages are activated to secrete IL-1β and IL-18, conducive to host defense and inducing a Th17 adaptive immune response (83). In addition, dsDNA has been found to trigger AIM2-ASC-dependent apoptosis dominated by caspase-1-induced pyroptosis in mouse macrophages (84). Disruption of Parkinson’s disease-associated mitochondrial serine protease HtrA2 activity leads to an increased response of the AIM2 inflammasome in macrophages (97). The above evidence also shows that AIM2 inflammasome activation in macrophages mediates immune responses in various infections.

Neutrophils are the first and most plentiful cells that permeate through the inflammatory region, where they can receive DAMPs (98) and then release many secretory vesicles containing proinflammatory factors and phagocytose pathogens to antagonize inflammation (99). Researchers have proven that neutrophils are an important source of IL-1β in both mice and human and that higher mRNA and protein levels of AIM2, ASC, and caspase-1 and AIM2 can induce GSDMD pyroptosis in neutrophils (85, 86). AIM2-mediated secretion of IL-1β upregulates IL-17 release by T cells of a murine model of atherosclerosis, which then drives chemokines such as CXC motif chemokine ligand 1 (CXCL1) and CXCL2 to recruit more neutrophils during inflammation (87).

## AIM2-inflammasome as a target for disease treatment

In recent years, targeted treatment of AIM2 has been proven successful in autoinflammatory diseases. Scientists have discovered that double-stranded RNA-dependent protein kinase (PKR) plays an important role in the activation of multiple inflammasomes, including the AIM2 inflammasome. Knockout of PKR or medicinal inhibitor application neutralizes inflammasome activation in mouse peritoneal macrophages under microbial infection and dsDNA trigger (100). Ahn et al. found that fructose-arginine (FA), a main component of Korean red ginseng, reduced the secretion of caspase-1, IL-1β, and IL-18 from mouse macrophages or human monocytes triggered AIM2 activation. They also demonstrated that FA could restrain the formation of ASC and the incision of GSDMD on the plasma membrane (101). Chung et al. reported that EFLA945, a constituent of red grape vine leaf extracts, prevents dsDNA from entering macrophages. Thus, EFLA945 reduces the oligomerization of ASC and inhibits the activation of caspase-1 and the release of the downstream cytokines IL-1β and IL-18. It is vital that EFLA945 also suppresses the mouse model immune response caused by IMQ (102). Liu et al. revealed that tripartite motif 11 (TRIM11) degrades AIM2 when *in vitro* cultured human macrophages or mice are infected with the virus and regulates the degradation of AIM2 through the autophagic cargo receptor p62 to mediate selective autophagy in macrophages (103). Dang et al. detected an interesting metabolic circuit between inflammasome activation and cellular cholesterol content regulation in mouse macrophages. Excess overabundance of cholesterol triggers the activation of AIM2. Therefore, macrophages activated by cholesterol could make use of cholesterol-25-hydroxylase (Ch25 h), an oxysterol that inhibits the synthesis of cholesterol, to restrain the AIM2 inflammasome from being activated (104).

## Whole-exome SNP array revealed *AIM2* in psoriasis

Our team has proven that *AIM2* is one of 15 new risk genes/loci of psoriasis through the whole-exome single nucleotide polymorphism (SNP) array for the first time. We used a large-scale whole-exome array and a three-stage case-control design to analyze the susceptibility gene of psoriasis, including 17,614 cases and 25,216 controls. Our meta-analysis of 76 psoriasis-related SNPs in the Exome Asian array and Exome Fine array and replication phase identified 11 variants in 11 different new susceptibility genes located in nonhuman leukocyte antigen (HLA) regions, including *AIM2* (rs2276405, P=3.22 × 10^−9^, odds ratio (OR) =0.83), which is a stop-gain gene variant locus (**Figure 1B**) (43). Even though GWAS have revealed more than 40 psoriasis susceptibility genes or loci, the majority of these genetic variants are located in noncoding genomic regions, and low frequency and rare genetic variants cannot be well explained; due to the small sample size, this method cannot detect some small-effect loci, the whole-exome SNP array can be a good complement to this deficiency (105–107).

## Epigenetic control of the *Aim2* in psoriasis

Our team reported that cg07195224 (**Figure 1B**), a CPG site of *AIM2*, located in the promoter region of the gene, is significantly hypomethylated in psoriatic lesions. The intensity of the promoter-associated peak is negatively correlated with the level of methylation at this CPG site and positively correlated with the level of *AIM2* mRNA expression. Meanwhile, our team identified Fra1 transcription factor (**Figure 1B**) target binds upstream of *AIM2* transcriptional start site, whose upregulation leads to AIM2 inflammasome activation. Binding of transcription factors may lead to DNA hypomethylation, thereby maintaining the open structural domains of chromatin, and increased chromatin accessibility is closely linked to psoriasis pathogenesis (108).

## Evidence of Aim2 inflammasome-related signals in psoriasis

The cGAS-STING is a DNA recognition signaling pathway that can induce programmed cell death, regulate the cell life cycle, prevent pathogen infections, and be involved in autoimmune or inflammatory diseases (109). When dsDNA in macrophages or keratinocytes is recognized by cGAS, it can activate the synthesis of cyclic GMP-AMP (cGAMP), and STING is activated by cGAMP induction in turn and triggers the downstream Interferon regulatory Factor 3 (IRF3)/IFN and NF-κB/IL-6 signaling cascades (110). The cGAS-STING signaling pathway activates AIM2 inflammasomes in two ways. Firstly, cGAMP is directly involved in the initiation and activation of cGAS-DNA induced inflammasomes, but STING not participates in this step and plays only a partial role in AIM2-dependent IL-1β secretion. Secondly, binding of cGAMP and STING upregulate the cytokines such as IFN-β and TNF, which are important signals in inflammasome components activation, then initiate and activate AIM2 inflammasome (111). Researchers found increased expression of STING and its downstream cytokines in skin lesions of psoriasis patients and the imiquimod (IMQ)-induced psoriasiform mouse model. Knockdown of STING reduced skin symptoms in a murine psoriasiform inflammation model, which might be related to the reduced IL-1β produced by keratinocytes (112). AIM2 inflammasome also inhibit cGAS-STING pathway by two methods. One is activated caspase-1 binds and cleaves cGAS directly, leads to the reduced cGAMP and downstream cytokine production (113). The other is caspase-1-activated GSDMD leads to potassium ion efflux from the membrane pore, which inhibits the activation of cGAS and cGAMP, then conduces to the reduced IFN-β production in turn (114). AIM2-deficient cells showed enhanced expression of cGAS-STING because the inhibition of AIM2 inflammasome pathway will reduce the pyroptosis, which leads to cGAMP and STING aggregation, as well as increased STING downstream components (115). IFN-β produced by cGAS-STING pathway also can activate the JAK-STAT pathway, cross-link AIM2 inflammasome and these signaling pathways (**Figure 1C**), mediate the development of psoriasis together.

## AIM2-mediated trained immunity in psoriasis

Former outlooks insisted that only adaptive immunity could built immunological memory, but recent studies have changed that point of view (116, 117). The immune systems of plants and invertebrates, as well as vertebrates, have the function of securing immune memory after the first stimulation (118) and reinforcing responses to protect from a secondary infection, a phenomenon called trained immunity or innate immune memory (119–121). Immune memory is triggered by the interaction of PRRs and PAMPs (120). Trained immunity, different from classical immunological memory, driven by signals of transcription factors and epigenetic reprogramming, does not induce permanent genetic changes (122, 123). Naik et al. described trained immunity in inflammation-experienced epithelial stem cells, and AIM2 and the downstream IL-1β played a key role in this immune response process (65), the first demonstration that trained immunity can be established not only by immune cells but also in nonimmune cells (**Figure 1H**).

## Trained immunity of cells may be associated with psoriasis

Stromal cells are defined as the heterogeneous cell group of mesenchymal origin, which includes fibroblasts, reticular stromal cells, and tissue-specific connective tissue cells such as osteoblasts and adipocytes (124, 125). The trait of secretory, immunomodulatory, and homing properties of these cells contributes to the function of supporting immune cells, building immune memory and immunoregulatory properties as well as antimicrobial activity (126–128). Mesenchymal stromal cells can regulate innate and adaptive immune responses *via* inflammatory cytokines released into the microenvironment (129). Lin et al. reported for the first time that when mesenchymal stromal cells were repeatedly exposed to lipopolysaccharide (LPS) in mice, the activation of NF-κB was increased significantly, enhancing the anti-inflammatory effect of macrophages, and this process was associated with histone methylation (130). Gingival fibroblasts have been found to maintain a low-grade inflammatory response until the second encounter with LPS and then respond rapidly to reduce inflammation after engagement (131). Synovial cells from patients with rheumatoid arthritis can display an inflammatory memory function similar to the inflammatory memory function of T cells. Endothelial cells also show an altered (rising or falling) inflammatory response after secondary stimulation (132).

Vascular endothelial cells continuously detect relevant danger signals such as pathogens, metabolic substances or cytokines in the blood, leading to an inflammatory response upon activation (133). The concept that vascular endothelial cells are conditioned innate immune cells was proposed by Mai et al. (134). Because vascular endothelial cells can experience epigenetic changes upon stimulation, leading to the generation of immune memory (135).

The group of 2 innate lymphoid cells (ILC2s) are regarded as tissue-resident lymphocytes. The ILC2s have been found to lack antigen-specific receptors, and thus memory ILC2s have no antigen specificity, which was considered to be consistent with the characteristics of innate immune memory (136). Skin ILC2s can participate in atopic dermatitis (AD), another chronic recurrent inflammatory disease, through activation by bacterial or other stimuli and then acquiring immune memory, leading to skin damage relapse (137, 138).

Monocytes are reputed to be the prophase of macrophages. When tissues suffer from infections, adult monocytes remove inflamed tissues and differentiate into macrophages that clear up the pathogen (139). Research has shown that when monocytes encounter *Candida albicans* (140) and *Bacille Calmette-Guérin* (BCG) vaccination (141), intensified protection against infections is induced, which is known as trained immunity. Trained immunity of monocytes/macrophages may be associated with the inflammasomes (139).

NK cells are innate lymphocytes that have shown the characteristic of adaptive immunity, in addition to the main feature of responding rapidly against pathogens and tumors in an antigen-independent manner (142). NK cells have been found to stimulate increased protective mechanisms against viral infections in mice after infection with influenza virus or cowpox virus (143), and there is also extensive evidence in primate disease models of viruses to demonstrate this stimulation (144). When NK cells are in a noninfected environment, they can be nonspecifically activated by proinflammatory cytokines and possess a stronger inflammatory response than resting NK cells when tested after a period of time (118, 145).

## Does the AIM2 inflammasome-induced trained immunity link to psoriasis recurrence?

Several studies in recent years that obtained startling results from a series of psoriasis modeling experiments in mice showed that skin resident memory T (TRM) cells (146), memory-like γδ T cells (147), keratinocytes and skin epithelial stem cells (EpSCs) (65) drive the relapse of psoriasis. When mouse skin exposed to psoriatic-like stimulation, EPSCs may remember the stimuli and ensures that the epidermis responds more rapidly and intensively to subsequent diverse challenges (65, 148). EpSCs are basal keratinocytes that obtain inflammatory memory in the background of psoriatic skin inflammation, and chromatin domains remain accessible for rapid activation and opening in response to secondary inflammatory stimulation (65, 149). The rapid and enhanced acquired inflammatory memory is particularly dependent on the AIM2 inflammasome and its downstream component IL-1β in EpSCs, and the induction of epidermal AIM2 expression to promote inflammatory repair could occur regardless of the activity of IMQ or other stimuli (65). Several studies have proven that inflammasomes and inflammasome-dependent IL-1β expression play an important role in immune memory (150, 151).

Numerous studies have shown that there is a strong correlation between trained immunity and epigenetic reprogramming, such as DNA methylation (152, 153). Previous work by our team has demonstrated increased chromatin accessibility in psoriatic lesions in patients with psoriasis. Most of the differential peaks in chromatin accessibility detected in psoriatic lesions overlapped with the peaks of DNase I hypersensitivity in keratinocytes, and more than 95% of psoriasis-associated CPGs found in psoriatic lesions were hypomethylated (108). Our team has demonstrated that cg07195224 is included in the promoter region of the AIM2 gene, which is the CPG site of significant hypomethylation in psoriasis (154).

With all that in mind, we can speculate that AIM2 might be the key marker modulating trained immunity in epidermal keratinocytes, as well as an important factor leading to the relapse of psoriasis, which needs more evidence for verification.

## Inflammasome-independent roles of AIM2 may related to psoriasis

**Figure 2:**
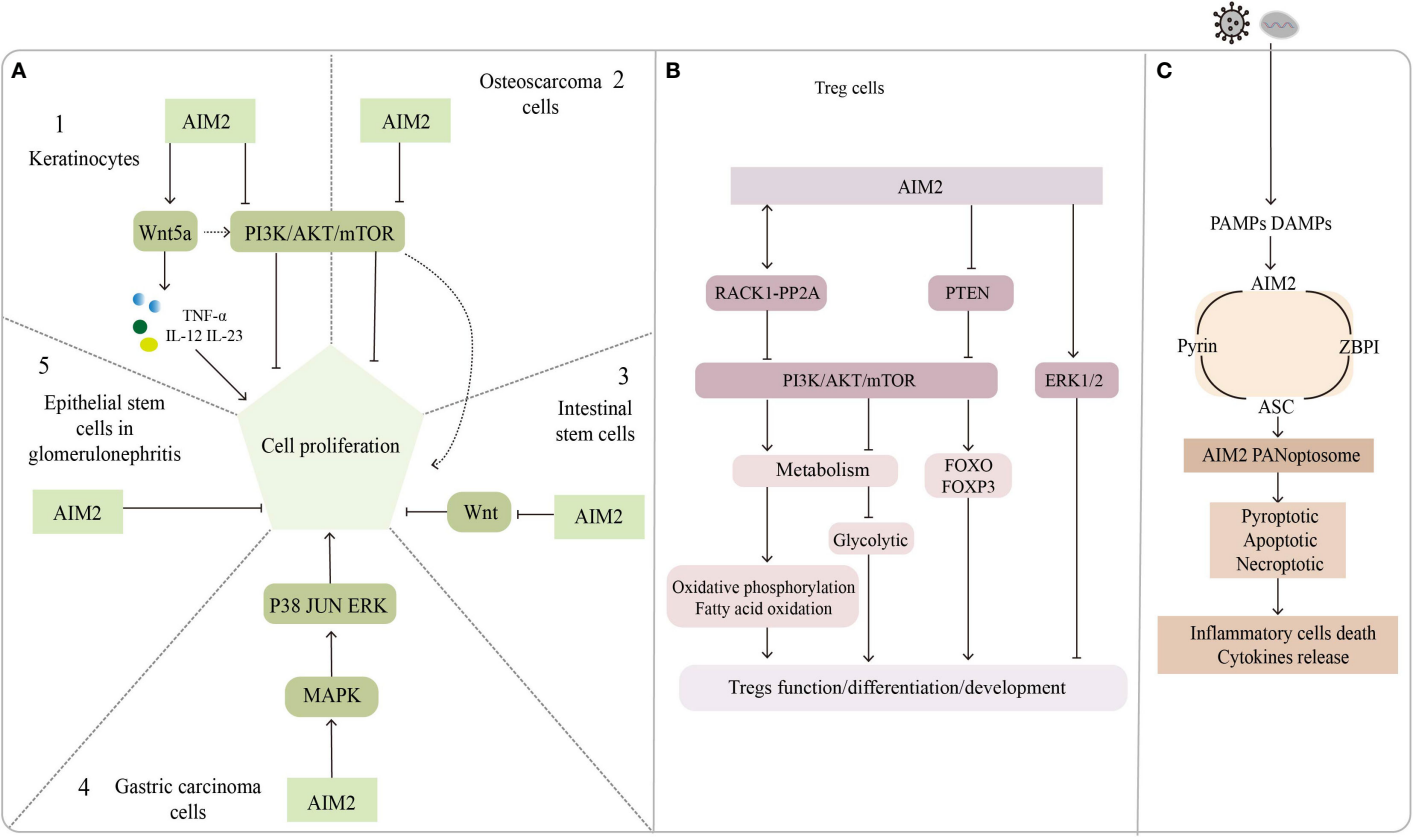
The inflammasome-independent way of AIM2. **(A)** Regulatory role of AIM2 in cell proliferation. A1, A2: AIM2 promotes keratinocytes proliferation through the Wnt5a pathway; PI3K/AKT pathway promotes keratinocytes and osteosarcoma cells proliferation, but AIM2 inhibits it. A3: AIM2 inhibits the proliferation of intestinal stem cells by suppressing the Wnt pathway, AIM2 activates PI3K/AKT pathway *via* Wnt5a and promotes intestinal stem cell proliferation. A4: AIM2 promotes gastric carcinoma cell proliferation through MAPK pathway. A5: AIM2 put a curb on epithelial stem cells proliferation in glomerulonephritis. **(B)** Role of AIM2 in Treg cells function/development/differentiation. AIM2 interacts with RACK1-PP2A protein to inhibit AKT/mTOR pathway, leading to metabolite changes that regulate Treg function and stability; AIM2 inhibits Treg proliferation through ERK1/2 pathway or by inhibiting PI3K/AKT pathway. **(C)** AIM2 forms a complex named AIM2 PANoptosome with pyrin, ASC and ZBPI, which mediates inflammatory cell death and cytokine release.

In addition to its critical role in the inflammasome and trained immunity, AIM2 has also been implicated in several other ways to modulate skin homeostasis, which might potentially be involved in psoriasis occurrence, development, or recurrence (**Figure 2**). For example, AIM2 might promote the proliferation of epithelial cells (155) and regulate the function of Treg cells (24); both of these cells are instrumental cell types that execute important functions in psoriasis development.

## AIM2 might disturb the proliferation of epithelial cells

Keratinocytes are a type of epithelial cell that might modulate psoriasis development by both directedly triggering an immune cascade and passively responding to cytokines when the skin microenvironment is altered. Several lines of evidence suggest that genetic modification of some genes might lead to psoriasis-like phenotypes but does not engage the systemic inflammatory response. For example, mice constitutively activating STAT3 in keratinocytes develop a psoriasis phenotype (156). Specific deletion of the NF-κB-responsive gene IκBa in keratinocytes leads to skin inflammation, including epithelial compartment and immune cell infiltration (157).

Recently, GWAS identified dozens of susceptible genes that might contribute to keratinocyte hyperproliferation and aberrant immune response (158). Some of these genes increased or decreased the risk of affecting the disease phenotypes when they were specifically deleted in keratinocytes. Keratinocyte-specific loss of function of target genes such as TNIP1, TNFAIP3, or CD109 in mice does not spontaneously develop visible skin inflammation, but when treated with IMQ, these mice were more likely to develop or show severe psoriasis symptoms than control mice (159–161). Meanwhile, JunB/c-Jun double knockout and N-WASP knockout in mice keratinocytes directly lead to hyperplasia and chronic inflammation (162, 163). In contrast, IL17RA and TRAF6 deletion in keratinocytes might protect mice from skin inflammation (164, 165). In summary, keratinocytes are key for psoriasis development, and their proliferation can be controlled by a single molecular or signaling pathway.

We noticed that most of these genes modulate psoriasis through the secretion of IL-23, and that downstream signaling molecules of the AIM2 inflammasome may act in conjunction with IL-23 to regulate psoriasis development, besides AIM2 might be directly involved in keratinocyte proliferation in an inflammasome-independent manner. AIM2 mutations have been found in 56% of small bowel cancers (166) but in only 2% of melanomas. Man and his colleagues found that AIM2-deficient mice presented uncontrolled proliferation of intestinal stem cells through an abnormal wingless (Wnt) signaling pathway, while inflammasome function was found to be undisrupted by assessing IL-1β, IL-18, and caspase-1 (155). Feng et al. reported that AIM2 might promote cell proliferation in gastric cancer *via* the mitogen-activated protein kinase (MAPK) pathway (167), leading us to suspect that AIM2 might also control keratinocyte proliferation by augmenting the Wnt or MAPK pathways. Indeed, Wnt5a increases keratinocyte proliferation by secreting TNF-α, IL-12, and IL-23 but repressing the Notch1 pathways which could activate the AIM2 inflammasome (168, 169). In contrast, AIM2 might repress proliferation by inactivating the phosphatidylinositol-3-kinase/protein kinase B/mammalian target of rapamycin (PI3K/AKT/mTOR) signaling pathway in osteosarcoma cells (170). Wilson et al. shown that AIM2 inhibits tumor cell proliferation by suppressing AKT phosphorylation in colonic epithelial cells and colon cancer cells, does not affect body inflammation and inflammasomes activation (171). PI3K/AKT signaling pathway also play an important role in the regulation of keratinocyte hyperproliferation in psoriasis (172), leading us to assume that AIM2 might regulate the proliferative state of keratinocytes in psoriasis by interacting with the PI3K/AKT signaling pathway, although the inhibitory effect of AIM2 is not very strong. The ability of AIM2 to inhibit cell proliferation was also found in glomerulonephritis (173). In summary, AIM2 can activate or inhibit cell proliferation *via* different signaling pathways. The exact role of AIM2 in modulating keratinocytes might be context-dependent.

## AIM2 might regulate the function of Treg cells in psoriasis

Treg cells are a kind of suppressive helper T cells that express high levels of IL-10 and the transcription factor FOXP3. By releasing TGF-β and IL-10, Treg cells can inhibit effector immune T cells, thus playing important roles in maintaining immunological self-tolerance and executing host defense immune responses in a healthy state (174, 175). Even though the percentage or frequency of circulating and infiltrating Tregs in psoriasis is controversial among studies (176, 177), impaired function has been confirmed by several groups; for example, the suppressive ability of Tregs was decreased, showing reduced CD73 expression and an inactive CD73/AMPK/mTOR pathway (178).

In psoriasis, Treg cell dysfunction can be lead to an imbalance in Th17/Treg cells, but the detailed mechanism underlying this phenomenon remains elusive (179). Recently, several epigenetic markers that cause imbalanced Th17/Treg cells have been identified in inflammatory psoriasis. Wu et al. found that miRNA-210 and miRNA-126 can promote the differentiation of Th17 cells, thus leading to an increased Th17/Treg ratio and psoriatic-like phenotypes in genetically engineered mice (180, 181). Ma and his colleagues found that the mis-regulated Notch1 signaling pathway increased the Th17/Treg ratio in psoriasis (182). In summary, impaired suppressive function of Tregs or an increased Th17/Treg ratio would cause psoriasis, mainly because these conditions can lead to an abnormal proinflammatory cytokine environment, such as upregulated IL-17A and IFN-γ and downregulated IL-10 and TGF-β.

AIM2 can regulate the development and function of Treg cells by regulating signaling pathways. The PI3K/AKT signaling pathway and its upstream or downstream components play critical roles in regulating Treg cell differentiation, development and function (183), and AIM2 was proved to suppress PI3K/AKT activation in an inflammasome-independent way (171). AIM2 in non-small cell lung cancer cells can promote extracellular signal-regulated kinase 1/2(ERK1/2) phosphorylation, the function has nothing with inflammasomes, while ERK1/2 was found to inhibit the development of FOXP3+ Treg cells (73).

Accumulating evidence suggests that metabolic processes are involved in the regulation of growth, differentiation, localization, and function of Treg cells (184). The regulatory effects of metabolic substrates such as lipids, amino acids, and glucose are dependent mainly on the cell state and the external environment and are largely flexible and dynamic. These metabolites have been found to contribute to the activation and maintenance function of Treg cells (185). FOXP3 is not only essential for Treg differentiation but also for regulating the expression of critical genes encoding metabolic molecules in Treg cells (186). For instance, *in vitro* analysis showed that FOXP3 activated the proliferation of Treg cells, which was inhibited by lactate (187). More recently, Chou et al. found that AIM2 promoted oxidative phosphorylation of lipids while suppressing aerobic glycolysis in Treg cells, an effect works in part by attenuating AKT phosphorylation and the mTOR and MYC signaling pathways, rather than by the activation of the AIM2 inflammasome, Treg stability is essential for regulating autoimmune diseases (24). These findings support the notion that regulation of metabolism is important for the suppressive capabilities of Treg cells and contributes to the maintenance of homeostasis at the skin barrier.

Chou’s finding attracted us to believe that regulation of Treg cell function through AIM2 can mediate the pathogenesis of psoriasis or other inflammatory diseases.

## AIM2 might control keratinocyte cell death

The AIM2 inflammasome could trigger the innate immune response by producing mature proinflammatory cytokines such as IL-18 and IL-1β, and it might also activate cell necroptosis when facing infections. Additionally, dead cells release more cytokines and inflammatory mediators, initiating the signaling cascade to eliminate infected cells or triggering chronic inflammation (188).

Recent findings suggested that necroptosis mediated by receptor-interacting protein kinase 1 (RIPK1), RIPK3, and mixed lineage kinase domain-like protein (MLKL) has been implicated in psoriasis development (189–191). The expression of RIPK1 is elevated in psoriatic lesions compared with no lesion skin and atopic dermatitis, but downregulated RIPK1 enhances tumor necrosis factor-related apoptosis-inducing ligand (TRAIL) signaling in psoriasis (192). Furthermore, RIPK1 potentially mediates the interactions between RIPK3 and Z-DNA binding protein 1 (ZBP1), thus preventing the activation of RIPK3 and the upstream kinase protein MLKL (193). Conditional knockout of RIPK1 in the epidermis could induce ZBP1-dependent necroptosis and skin inflammation (194, 195). Recently, Lee and colleagues described a new multiprotein complex, named PANoptosis, which could detect and respond to herpes simplex virus 1 infection. In this study, the author found that AIM2 regulates the innate sensors pyrin and ZBP1 to drive inflammatory signaling and trigger cell death, which is characterized by the activation of critical pyroptotic, apoptotic and necroptotic molecules. Since AIM2 interacts with pyrin, ZBP1 and ASC to form the complex, they called it the AIM2 PANoptosome (196). Even though whether AIM2 PANoptosome plays roles in psoriasis has not been determined, we still think AIM2 might be a good target for drug development in the future.

## Conclusions and perspectives

Psoriasis is a chronic inflammatory skin disease that is closely related to many complications and seriously affects the appearance and mental health of patients. Treatment modalities for psoriasis include traditional oral drugs, topical medication, and phototherapy. Many oral or topical medications can have some negative effects on the body after long-term use (197). In recent years, biological agents have gradually become the first-line treatment for moderate to severe psoriasis (198). Although biologics anticytokines and antiproinflammatory pathways are highly targeted and effective, they also have disadvantages such as high price, leading to reduced immunity of patients, easy infection, and easy loss of efficacy, so there is still a need to develop new effective biological agents for personalized treatment of psoriasis (3). In recent years, AIM2 has been known as an important component in the pathogenesis of psoriasis, but whether AIM2 could be the next effective drug target for psoriasis is not yet clear. Inhibitors of AIM2 or molecules such as ASC and caspase-1 in the AIM2 inflammasome pathway have also been shown to be effective in cancers and some other diseases (199, 200). The downstream proinflammatory cytokines of the AIM2 inflammasome, IL-1β, and IL-18, can activate inflammatory cascade responses in psoriatic skin, such as the IL-23/Th17/IL-17 signaling axis, there are several corresponding targeted drugs for which there are currently a large number of successful cases in the treatment of psoriasis (201). However, the mechanisms by which AIM2 causes psoriasis involve the activation of various cytokines, proinflammatory pathways, and several signaling pathways in cells that are still not fully understood. There is also no direct evidence to show that AIM2 can regulate the progression of psoriasis by regulating the proliferation, metabolism, and apoptosis of related cells or by mediating trained immunity to regulate the recurrence of psoriasis. Moreover, the function of AIM2 in adaptive immune cells is also not fully understood. Therefore, we can only expect more research on these interesting mechanisms in the future.

## Author contributions

FZ proposed the idea of the paper and contributed to the review of this work with HC, YZ conceived and wrote the article, and XX made constructive revisions to the article. All authors contributed to the article and approved the submitted version.
